# A Four-Dimensional Volumetric Quantification of the Left Ventricle in Healthy Pregnant Women in the Third Trimester

**DOI:** 10.7759/cureus.46342

**Published:** 2023-10-01

**Authors:** Hanan Mohsen Ali Al-Allak, Asaad Hasan Noaman Al-Aboodi

**Affiliations:** 1 Department of Physiology, Faculty of Medicine, Al-Furat Teaching Hospital, Najaf, IRQ

**Keywords:** physiology, cardiac, third trimester pregnancy, left ventricular volume, four-dimensional echocardiography

## Abstract

Background: Hemodynamic load and heart structural remodeling rise during pregnancy because these changes are physiologically necessary. Adaptations in the mother's circulatory system may either initiate or aggravate the development of cardiovascular disease in the offspring. If the body is unable to adjust to these changes, it may develop heart conditions like cardiomyopathy. There is a lack of third-trimester echocardiographic data on left ventricular (LV) volume and function in healthy Iraqi women. To understand the cardiac alterations that occur during normal pregnancy, a precise tool that evaluates cardiac function is needed. In that regard, the four-dimensional echocardiography (4DE) technique has markedly improved the quality and accuracy of assessing the size and function of the left ventricle.

Aim: The present study aimed to assess LV volume and function in the third trimester of a healthy pregnancy using 4DE and to compare the results of LV assessment using 4DE with those of LV assessment using conventional two-dimensional (2D) echocardiography.

Methods: The study was conducted on 75 healthy pregnant women (the case group) and 75 non-pregnant women (the control group). The participants attended Al-Fortat Teaching Hospital from April 1, 2022, to May 30, 2023, and had 2D and 4D echocardiographic studies performed on them.

Results: The LV end-diastolic volume (EDV), end-systolic volume (ESV), and cardiac output (CO) were significantly increased in the case group compared to the control group (90.87 ± 18.03 ml vs. 62.64 ± 14.11 ml, P<0.001; 35.59 ± 6.52 ml vs. 22.42 ± 5.82 ml, P<0.001; and 4.87 ± 1.27 vs. 3.35 ± 0.87 L/m, P<0.001, respectively). In contrast, the LV ejection fraction (LVEF) was significantly decreased in the pregnant group compared to the control group (60.37 ± 5.42 % vs. 64.04 ± 4.99 %, P<0.01). Additionally, the study showed significant differences in EDV, ESV, ejection fraction (EF%), and CO (P<0.001) between 2D and 4D echocardiography, according to the Bland Altman test.

Conclusion: In healthy pregnant women in their third trimester, there is an increase in the indicators of preload (ventricular volume and CO) and a decrease in EF%. The 4DE provides detailed images and information about cardiac volumes and function, allowing for the early detection of any potential problems that may arise during pregnancy and thus improving the health outcomes of both the mother and the developing fetus.

## Introduction

In women, heart disease is the top killer. Mothers are expected to have some kind of cardiovascular disease in 1-4% of all pregnancies, making it the leading non-obstetric cause of maternal mortality [1,2]. Pregnancy has been referred to as a cardiovascular "physiologic stress test." Maternal cardiovascular adaptation can provoke new-onset cardiovascular disease or exacerbate a previously silent one [3].

During pregnancy, the total blood volume increases by about 45% above the pre-pregnancy volume, and red blood cell production increases by up to 40% via erythropoiesis [4]. Hemodilution causes "physiological anemia" because the plasma volume rises at a faster rate than the red blood cell mass [5]. Cardiac output (CO) increases by about 30-35% above pre-pregnancy levels due to cardiac contractility enhancement and stroke volume (SV) increase, which are caused by an increase in end-diastolic volume (EDV) [6]. The overall heart rate increases by 20-25% above the pre-pregnancy rate. Around the time the woman is in the middle of her second trimester, her peripheral vascular resistance begins to drop significantly and then remains relatively constant for the balance of her pregnancy. The elasticity of the blood vessels also improves.

There are several effects of these alterations on the mother's heart. Heart remodeling (increase in left ventricular [LV] wall thickness, LV cavity size, and LV mass) occurs in tandem with the reduction in systemic vascular resistance, which reduces LV hemodynamic afterload. The rise in blood volume also raises venous preload [7]. Inducing antiangiogenic factors and experiencing an abnormal reaction to hemodynamic stress may both cause and exacerbate heart disorders like peripartum cardiomyopathy [8]. It accounts for the bulk of maternal deaths not related to obstetric complications. Since LV volume and function are the most impacted parameters during pregnancy, it is essential to have a precise instrument to evaluate cardiac function and research these areas in order to fully grasp how pregnancy might lead to heart disease.

Because of its general availability and safety, echocardiography is the recommended diagnostic technique for the examination of structural, functional, and hemodynamic changes during pregnancy. Variations in ventricular loading circumstances occur during pregnancy, limiting the use of the standard indices of myocardial systolic function, such as ejection fraction (EF), shortening fraction, and tissue Doppler velocity, all of which are load-dependent [9]. Four-dimensional echocardiography (4DE) is closely related to the functional state of the LV and is a more sensitive tool for detecting the early, subtle changes in LV systolic function that occur in pregnant women before they become overt on conventional echocardiography and tissue Doppler imaging [10].

## Materials and methods

The study samples were collected from the gynecological consultation unit of Al-Furat Teaching Hospital in Al-Najaf Governorate from the beginning of April 2022 to the end of March 2023. The study included 75 healthy pregnant women in their third trimester (18-40 years old) and 75 healthy non-pregnant young women of the same age group, which were used as the control group. All subjects had a complete medical history obtained and were examined physically to rule out illnesses including organic heart disease, kidney disease, thyroid disease, severe anemia, diabetes, hypertension, and chronic medication or smoking. The height (cm) and weight (kg) of the women were obtained using a measuring tape and a digital weight scale, respectively. The body mass index of each woman was calculated as weight (kg)/height (m^2^) [11]. ECG and Abd U/S, to calculate gestational age, have been recorded. All participants in the study gave their informed consent before participating. Ethical numebr was IEC/2022/232. 

Echocardiographic analysis

All of the ladies had a full 2D and 4D ultrasound utilizing commercially accessible Vivid E9 ultrasound equipment (GE Healthcare, Horten, Norway) with a 2.5 MHz S5-1 transducer. As suggested by the American Society of Echocardiography, the ladies were assessed with the echocardiography leads placed across the chest while lying in the left lateral decubitus position, which brings the heart closer to the chest wall and laterally to the sternum [12].

To acquire a parasternal long-axis view for the LV ejection fraction measurement, the transducer was positioned in the third intercostal space with the indicator pointing toward the right shoulder, as in 2D-guided M-mode echocardiography. Each and every purchase was handled by the same seasoned professional. We used a commercially available, previously validated semi-automated technique called 4D auto LV volume quantification (4DLVQ, EchoPAC PC version 108.1.4, GE Healthcare) to analyze the data sets digitally after they had been collected and saved off-line [13].

Automatically presented in quad-view (apical four-chamber, two-chamber, three-chamber, and LV short-axis plane) were the end-diastolic (ED) frames used for contour identification. First, the four-chamber plane had to be manually adjusted such that the correct intersecting line of all the planes passed through the exact middle of the LV cavity, spanning the LV apex and the mitral valve opening. When using the program, two points must be placed at the mitral annulus limits and one point must be placed at the apex in end diastolic (ED) frames and then in end systolic (ES) frames. The LV endocardial boundary is drawn in a 3D model automatically during the ED and ES phases. If the endocardial boundary was not properly drawn by the computer, the boundaries were changed by hand. Finally, the left ventricular (LV) systolic volume, end-diastolic volume, end-systolic volume in four dimensions, and EF were shown [13].

Statistical analysis

The research was quantitative, and SPSS version 18 was used to analyze the data. Both the whole bunch I (n = 75) and a control group (n = 75) were used to analyze the data. The review groups were examined using a free T-test, and the mean, standard deviation, and standard blunder of the mean were calculated. The Boring Altman study was used to compare the accuracy of 2D and 4D echocardiography, and a significance level of 0.05 was deemed necessary.

## Results

Four-dimensional LVEDV

This study showed that the mean 4D EDV in the pregnant women group was 90.87 ± 18.03 ml; this result was significantly higher (p<0.001) than that of the non-pregnant women group (62.64 ± 14.11 ml).

Four-dimensional LVESV

Our study revealed that the mean 4D ESV of the pregnant group was 35.59 ± 6.52 ml, which was significantly (p<0.001) higher than that of the non-pregnant women's group (22.42 ± 5.82 ml) (Table 1).

**Table 1:** The distribution of 4DE variables in the pregnant and non-pregnant groups of women

Variable	Pregnant women (n=75) mean ± SD	Non pregnant women (n=75) mean ± SD	P-value
4D EDV (ml)	90.87 ± 18.03	62.64 ± 14.11	0.001
4D ESV (ml)	35.59 ± 6.52	22.42 ± 5.82	0.001
4D EF%	60.37 ± 5.42	64.04 ± 4.99	0.001
4D CO (L/min)	4.87 ± 1.27	3.35 ± 0.87	0.001

Four-dimensional EF%

The mean 4D EF% for the systolic function was lower in the pregnant women group (60.37 ± 5.42%) compared with the non-pregnant women group (64.04 ± 4.99%); therefore, pregnancy was independently and significantly associated with a lower EF% (p<0.01).

Four-dimensional cardiac output (4D CO)

The mean 4D CO of the pregnant group (4.87 ± 1.27 L/m) was significantly higher (p<0.001) than that of the non-pregnant group (3.35 ± 0.87 L/m). The distribution of 2DE variables in the pregnant and non-pregnant groups showed a significant difference (Table 2).

**Table 2:** The distribution of 2D echocardiographic variables in the pregnant and non-pregnant groups of women

Variable	Pregnant (n=75) mean ± SD	Control (n=75) mean ± SD	P-value
2D EDV (ml)	117.73 ± 23.16	85.95 ± 24.96	0.0001
2D ESV (ml)	40.96 ± 10.72	27.85 ± 7.45	0.0001
2D EF%	65.69 ± 7.08	69.98 ± 6.25	0.0001
2D CO L/min	6.7 ± 2.1	5.1 ± 2.7	0.0001

## Discussion

It is well known that pregnancy puts a heavy burden on the heart because of the hormonal changes that occur in the mother's circulatory system. The present research demonstrated a statistically significant increase in 4D LVEDV during pregnancy. In comparison to the control group, all measurements were considerably higher in pregnant women. Another study found that the LVEDV, as assessed by regular echocardiography, is noticeably higher than normal during pregnancy [14]. Because cardiac adaptations in pregnant women are thought to begin in the first few weeks of pregnancy, this may make sense. Due to the increased blood volume, preload is increased, and this is shown as an enhanced LVEDV.

Mesa et al. [15] described, using echocardiography, an increase in LVEDV as early as 10 weeks gestation and a peak during the third trimester, whereas Geva et al. [16] found no change in LV EDV or ESV during the course of the pregnancy. Compared to the control group, pregnant women in our research had considerably higher 4D LVESV. Using traditional echocardiography, several investigators have also shown a considerable rise in LVESV during pregnancy [17].

Table 1 shows that, compared to the non-pregnant group, the EF% was considerably lower in the pregnant group (p=0.001). This finding is consistent with another study showing a considerable drop in EF% in the second and third trimesters of pregnancy [9]. Another study showed that LVEF dropped somewhat during the second trimester but recovered during the third [17]. Another study found no significant change in LVEF during pregnancy [15,16], contrary to an earlier report by Hunter and Robson [18], who discovered an improvement in LVEF over the first two trimesters of pregnancy. These results provide further evidence that there is ethnic variation in the cardiovascular response to a healthy pregnancy.

When comparing pregnant women and controls, we discovered that CO (4D CO) levels were significantly higher in the pregnant group (p = 0.001). Different studies have shown either an increase, a decrease, or no change in CO during the third trimester, and this discrepancy has been attributed mainly to the mother's adaptations throughout pregnancy as well as the effects of anthropometric variables and body positioning [19]. Although many studies have demonstrated that CO increases by 30-35% above the pre-gestational level (6), a 30-50% increase in cardiac output can ultimately occur [20].

Furthermore, in the pregnant women's group, 2D EDV (117.73 ± 23.16) was significantly higher than 4D EDV (90.87 ± 18.03) and 2D ESV (40.96 ± 10.72), which in turn was significantly higher than 4D ESV (35.59 ± 6.52). The same is true for CO, which had significantly higher values on 2DE (6.7 ± 2.1) than on 4DE (4.87 ± 1.27); this is attributed to the fact that the ventricular volumes and CO (which are both indicators of preload) increased progressively during pregnancy [21]. The ventricular chambers may expand in response to a rise in blood volume, although non-pregnant individuals often fall within the normal range for ventricular characteristics [22].

The LVEF% is generally unchanged or slightly increased during pregnancy. Currently, the data are conflicting [22,23], but we found the 2D EF% to be significantly higher (65.69 ± 7.08) than the 4D-AutLVQ values (60.37 ± 5.42). The left ventricle is seen ''as it is'' in four-dimensional echocardiography, unaffected by loading situations like pregnancy. Also, it compares well to cardiac magnetic resonance (CMR) in terms of volume and ejection fraction (EF%), and its 4D repeatability is on par with CMR's [24,25].

Volumes of LVs are estimated in M-Mode; however, this method has several problems since it assumes the LV is a prolate ellipse and that volume can be determined by measuring a single axis dimension and cubing it. Errors grow exponentially with the number of measurements, leading M-Mode and 2DE to incorrectly assume the geometric properties of the left ventricle [25].

## Conclusions

In healthy pregnant women in the third trimester, there is an increase in the indicators of preload, ventricular volumes (ESV and EDV), and cardiac output and a decrease in EF%. Four-dimensional echocardiography provides detailed images and information about the cardiac volumes and function, allowing for the early detection of any potential problems that may arise during pregnancy and hence improving the health outcomes of both the mother and the developing fetus.

## References

[1] Brown HL, Smith GN (2020). Pregnancy complications, cardiovascular risk factors, and future heart disease. Obstet Gynecol Clin North Am.

[2] Aggarwal N, Wood M (2021). Sex Differences in Cardiac Disease. https://shop.elsevier.com/books/sex-differences-in-cardiac-diseases/aggarwal/978-0-12-819369-3.

[3] Ramlakhan KP, Johnson MR, Roos-Hesselink JW (2020). Pregnancy and cardiovascular disease. Nat Rev Cardiol.

[4] Mehta LS, Warnes CA, Bradley E (2020). Cardiovascular considerations in caring for pregnant patients: a Scientific Statement From the American Heart Association. Circulation.

[5] Afari HA, Davis EF, Sarma AA (2021). Echocardiography for the pregnant heart. Curr Treat Options Cardiovasc Med.

[6] Adeyeye VO, Balogun MO, Adebayo RA (2016). Echocardiographic assessment of cardiac changes during normal pregnancy among Nigerians. Clin Med Insights Cardiol.

[7] Ando T, Kaur R, Holmes AA, Brusati A, Fujikura K, Taub CC (2015). Physiological adaptation of the left ventricle during the second and third trimesters of a healthy pregnancy: a speckle tracking echocardiography study. Am J Cardiovasc Dis.

[8] Bauersachs J, König T, van der Meer P (2019). Pathophysiology, diagnosis and management of peripartum cardiomyopathy: a position statement from the Heart Failure Association of the European Society of Cardiology Study Group on peripartum cardiomyopathy. Eur J Heart Fail.

[9] Cong J, Fan T, Yang X, Squires JW, Cheng G, Zhang L, Zhang Z (2015). Structural and functional changes in maternal left ventricle during pregnancy: a three-dimensional speckle-tracking echocardiography study. Cardiovasc Ultrasound.

[10] Eroğlu AG, Yüksel EK, Karagözlü F, Acar HC, Gökalp S, Evliyaoğlu O (2023). Evaluation of the left ventricular systolic function and myocardial deformation by real-time three-dimensional (four-dimensional) and speckle-tracking echocardiography in children with type 1 diabetes mellitus. Cardiol Young.

[11] Addo VN (2010). Body mass index, weight gain during pregnancy and obstetric outcomes. Ghana Med J.

[12] Lang RM, Badano LP, Mor-Avi V (2015). Recommendations for cardiac chamber quantification by echocardiography in adults: an update from the American Society of Echocardiography and the European Association of Cardiovascular Imaging. J Am Soc Echocardiogr.

[13] Shahgaldi K, Manouras A, Abrahamsson A, Gudmundsson P, Brodin LA, Winter R (2010). Three-dimensional echocardiography using single-heartbeat modality decreases variability in measuring left ventricular volumes and function in comparison to four-beat technique in atrial fibrillation. Cardiovasc Ultrasound.

[14] Hall ME, George EM, Granger JP (2011). [The heart during pregnancy]. Rev Esp Cardiol.

[15] Mesa A, Jessurun C, Hernandez A, Adam K, Brown D, Vaughn WK, Wilansky S (1999). Left ventricular diastolic function in normal human pregnancy. Circulation.

[16] Geva T, Mauer MB, Striker L, Kirshon B, Pivarnik JM (1997). Effects of physiologic load of pregnancy on left ventricular contractility and remodeling. Am Heart J.

[17] Sengupta SP, Bansal M, Hofstra L, Sengupta PP, Narula J (2017). Gestational changes in left ventricular myocardial contractile function: new insights from two-dimensional speckle tracking echocardiography. Int J Cardiovasc Imaging.

[18] Hunter S, Robson SC (1992). Adaptation of the maternal heart in pregnancy. Br Heart J.

[19] Desai DK, Moodley J, Naidoo DP (2004). Echocardiographic assessment of cardiovascular hemodynamics in normal pregnancy. Obstet Gynecol.

[20] Shiina Y (2019). Maternal and fetal cardiovascular disease. Cardiovascular Assessment During Pregnancy.

[21] Savu O, Jurcuţ R, Giuşcă S (2012). Morphological and functional adaptation of the maternal heart during pregnancy. Circ Cardiovasc Imaging.

[22] O'Kelly AC, Sharma G, Vaught AJ, Zakaria S (2019). The use of echocardiography and advanced cardiac ultrasonography during pregnancy. Curr Treat Options Cardiovasc Med.

[23] Liu S, Elkayam U, Naqvi TZ (2016). Echocardiography in pregnancy: part 1. Curr Cardiol Rep.

[24] Bu L, Munns S, Zhang H (2005). Rapid full volume data acquisition by real-time 3-dimensional echocardiography for assessment of left ventricular indexes in children: a validation study compared with magnetic resonance imaging. J Am Soc Echocardiogr.

[25] Monaghan MJ (2006). Role of real time 3D echocardiography in evaluating the left ventricle. Heart.

